# What Do Patients Complain About Online: A Systematic Review and Taxonomy Framework Based on Patient Centeredness

**DOI:** 10.2196/14634

**Published:** 2019-08-07

**Authors:** Jing Liu, Shengchao Hou, Richard Evans, Chenxi Xia, Weidong Xia, Jingdong Ma

**Affiliations:** 1 School of Medicine and Health Management Tongji Medical College Huazhong University of Science and Technology Wuhan China; 2 Library, Tongji Hospital Tongji Medical College Huazhong University of Science and Technology Wuhan China; 3 College of Engineering, Design and Physical Sciences Brunel University London London United Kingdom; 4 Department of Information Systems and Business Analytics College of Business Florida International University Miami, FL United States

**Keywords:** patient-centered care, delivery of health care, systematic review, taxonomy

## Abstract

**Background:**

Complaints made online by patients about their health care experiences are becoming prevalent because of widespread worldwide internet connectivity. An a priori framework, based on patient centeredness, may be useful in identifying the types of issues patients complain about online across multiple settings. It may also assist in examining whether the determinants of patient-centered care (PCC) mirror the determinants of patient experiences.

**Objective:**

The objective of our study was to develop a taxonomy framework for patient complaints online based on patient centeredness and to examine whether the determinants of PCC mirror the determinants of patient experiences.

**Methods:**

First, the best fit framework synthesis technique was applied to develop the proposed a priori framework. Second, electronic databases, including Web of Science, Scopus, and PubMed, were searched for articles published between 2000 and June 2018. Studies were only included if they collected primary quantitative data on patients’ online complaints. Third, a deductive and inductive thematic analysis approach was adopted to code the themes of recognized complaints into the framework.

**Results:**

In total, 17 studies from 5 countries were included in this study. Patient complaint online taxonomies and theme terms varied. According to our framework, patients expressed most dissatisfaction with *patient-centered processes* (101,586/204,363, 49.71%), followed by *prerequisites* (appropriate skills and knowledge of physicians; 50,563, 24.74%) and *the care environment* (48,563/204,363, 23.76%). The least dissatisfied theme was *expected outcomes* (3651/204,363, 1.79%). People expressed little dissatisfaction with *expanded* PCC dimensions, such as *involvement of family and friends* (591/204,363, 0.29%). Variation in the concerns across different countries’ patients were also observed.

**Conclusions:**

Online complaints made by patients are of major value to health care providers, regulatory bodies, and patients themselves. Our PCC framework can be applied to analyze them under a wide range of conditions, treatments, and countries. This review has shown significant heterogeneity of patients’ online complaints across different countries.

## Introduction

As internet availability and usage grows worldwide, patients are spontaneously rating their experiences with physicians and hospitals by sharing their opinions about health encounters on the World Wide Web via mediums such as social media websites, Web-based consumer opinion platforms, and physician rating websites (PRWs) [1-5]. Previous research has demonstrated that patients are often influenced by peer-submitted comments posted on opinion and rating websites when making health care decisions [6,7]. On the basis of this notion, medical providers are able to leverage the information posted on such platforms to better comprehend patient experiences and engagement levels [4] and increase the understanding of patient frustrations and joy points during hospital visits [8-16]. By capturing patient data in real time, health care providers can use them as a quality metric to highlight insufficient physician performances or irregular events [5,17]. On the basis of extensive circumstantial evidence [18-20], countries such as the United Kingdom systematically collect data relating to patient experiences from their quality-reporting website (National Health Service, NHS choices) to support the further development of patient-centered care (PCC) [8,21-23].

Given the intrinsic value of comments posted online by patients, it is important that health care providers make efficient use of the information collected. Practices observed from the adverse event taxonomy proposed by Harrison et al [24], which facilitated the collection and aggregation of data to compare findings, identify priorities, and develop wide-reaching patient safety solutions, demonstrated the momentousness of a unified, agreed framework with standardized concepts and terms. Despite the large volume of work published in this domain (eg, the studies by Reader et al [25] and Li et al [26]), currently available taxonomies for analyzing online complaints made by patients often lack standardized themes, terminology, and underlying unifying theory, creating difficulties in making sense of data that cannot be used to compare against other services, organizations, or countries. An operational and rigorous framework that classifies complaints made by patients, containing standardized concepts with agreed definitions and preferred terminology and establishing the relationships between concepts based on an explicit and nonoverlapping domain ontology, is required [24].

When we take into consideration the well-developed *PCC framework*, which forms the basis for patient experience measurement systems in the United States, the United Kingdom, and other parts of Europe [27,28], we can see that it includes clear and proven terminologies with standardized dimensions and concepts. To create a patient-centric health care system that meets the needs and preferences of patients is one of the primary goals of numerous countries [17,21,27,29-32]. Therefore, it is feasible that we use the principles of PCC to guide the analysis of online complaints and examine whether the determinants of PCC are the same as the patient experience. To confirm this approach, a literature search was completed using a combination of keywords and subject headings, based on the defined concepts of patient complaints and PCC. Through analysis of the search results, it was identified that no study is yet to be completed that categorizes issues based on patient centeredness and that a generic taxonomy is required that appropriately analyzes issues against a wide range of conditions and in the context of different health care settings. These findings led to the following research questions (RQs) being posed:

- RQ1: Have previous studies formed or adopted a credible taxonomy framework?
- RQ2: Are available frameworks based on patient centeredness commensurate with what patients currently complain about online?
- RQ3: Which dimensions of PCC constitute the focus of online complaints made by patients?
- RQ4: Could a taxonomy framework allow us to identify the differences in patient complaints in a multicountry context?

To answer these questions, a systematic review approach was used. First, we followed a process of synthesis [29] to propose our a priori framework. Then, comprehensive searches were conducted to systematically identify qualified studies relating to patient complaints online; at this point, data were extracted to match with the a priori framework; those that matched were compared between countries.

**Figure 1:**
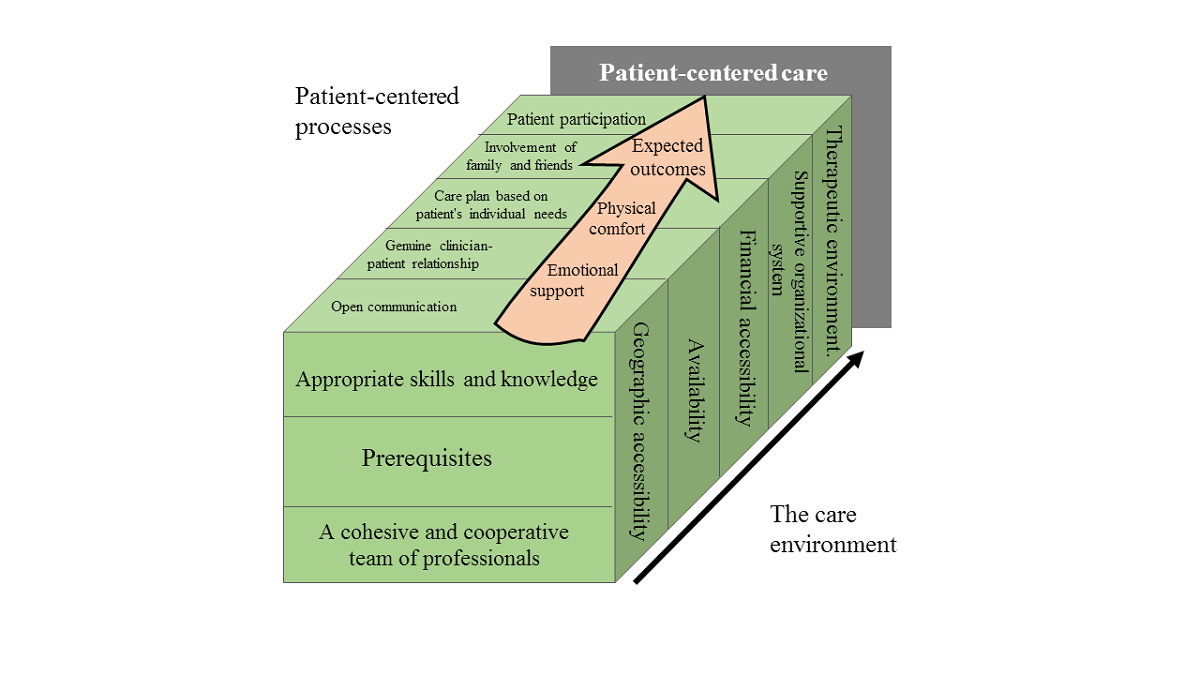
Proposed an a priori framework of online complaints based on patient centeredness.

To create the proposed framework, we synthesized all studies relating to PCC using the best fit framework synthesis technique proposed by Booth and Carroll [29]. First, we identified all the relevant frameworks or conceptual models that related to PCC, which are published in academic literature. At the forefront is the widely understood *Picker Principles of Care* framework, an internationally renowned approach used for measuring quality improvement in health care in the United States and the United Kingdom. Designed by the UK-based Institute for Healthcare Improvement, the PCC framework includes 8 dimensions: (1) respect for patient values, preferences, and expressed needs; (2) coordination and integration of care; (3) information and education; (4) physical comfort; (5) emotional support and alleviation of fear and anxiety; (6) involvement of family and friends; (7) continuity and transition; and (8) access to care. Although well adopted, the framework is considered a single-layer structure, which may lead to inefficiency in identifying homogeneous underlying problems. Brendan McCormack et al [33] developed a patient-centered framework comprising 4 constructs—prerequisites, the care environment, patient-centered processes, and expected outcomes—which was derived from Donabedian’s [34] structure-process-outcome assessment model. The proposed framework has been rigorously developed and tested in acute hospital settings [33] and is comprehensive enough to incorporate PCC dimensions. Second, we identified all relevant publications relating to the dimensions of PCC. Aside from the frameworks mentioned above, Kitson et al [35] and Rathert et al [36] completed systematic reviews of the PCC field and synthesized the common core elements of PCC. These 2 studies are highly cited and have been validated by a variety of follow-up studies. We compared and synthesized the dimensions of these 5 models. Third, we conducted a framework synthesis using thematic analysis [37]. Finally, grounded in the above, we developed an a priori framework with 4 domains, 8 categories, and 25 subcategories of online health care complaints based on patient centeredness, as shown in Figure 1.

In the first layer of the framework, the prerequisites focus on health care professionals having appropriate skills and knowledge and the team of professionals being cohesive and cooperative. The care environment refers to the context in which care is delivered and includes supportive organizational systems and accessibility, in terms of geography, financial affordability, and availability. Patient-centered processes focus on delivering care through a range of activities that operationalize person-centered care. Expected outcomes relate to the results expected from effective PCC, addressing a patients’ physical and emotional needs.

## Methods

The systematic review reported on hereafter was conducted according to the Preferred Reporting Items for Systematic reviews and Meta-Analyses guidelines[38]. As Reader et al [25] have previously systematically reviewed patient complaints, this study adopted some of their reporting items.

### Search Strategy

The electronic databases of ISI Web of Science, Scopus, and PubMed were searched for articles published between 2000 (because of the explosive growth of internet usage around 2000 [30]) and June 2018. A medical librarian (SCH) developed a Boolean search strategy. Then, a doctoral student (JL) carried out the search strategy, which was revised, if required, by SCH. The keywords searched in the *Title* or *Abstract* fields related to (1) *complaints* (eg, comments OR ratings OR suggestions OR reviews OR feedback) and (2) *online* (eg, free text OR social media OR e-health OR virtual OR internet OR Facebook OR twitter), which were subject to inquiry (see Multimedia Appendix 1).

### Inclusion Criteria

Studies were considered eligible if they were (a) related to the collection of primary quantitative data about patient complaints; (b) submitted by patients or third parties on their behalf; (c) comments uploaded to PRWs, organization’s websites, or any other online channel; or (d) conveyed in English to facilitate cross-country comparison.

### Exclusion Criteria

Studies considered ineligible from this research included those that only referred to (a) physician ratings, (b) satisfaction questionnaires, (c) complaints made to non–health care–related organizations, or (d) qualitative analysis without quantifiable themes.

### Study Selection

**Figure 2:**
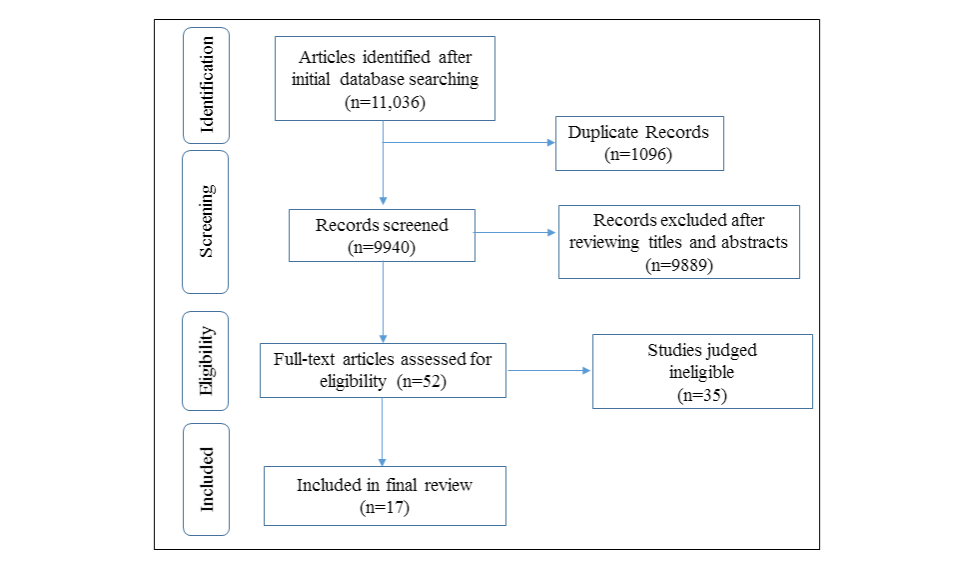
Preferred Reporting Items for Systematic reviews and Meta-Analyses flow chart.

After removal of duplicates, JL screened the titles and abstracts of all the remaining records for relevance. In the next stage, the full-article text of the retrieved results was independently examined by JL and SCH for inclusion. Discrepancies were adjudicated by a senior researcher (JDM). A total of 17 papers were included in this systematic analysis, as illustrated in Figure 2.

### Data Extraction and Analysis

Data were initially extracted by JL and subsequently checked by SCH, JL, and JDM, who together carried out the coding phase. The process consisted of the following phases: (1) descriptive and methodological data included in the study were extracted based on the items listed in the People-Centered and Integrated Health Services [33], (2) the number and categories of patient complaints were extracted, and (3) all themes or categories in selected studies were traversed and classified to match the initial categories. Data was analyzed using a thematic analysis approach adapted from the procedure outlined by Braun et al [37]. JL and SCH co-coded the issues of 2 papers to ensure that the coding framework and themes were commonly understood by the research team. JL and SCH independently coded the issues of the remaining 15 papers. In this paper, we achieved high interrater reliability (kappa=.82). Throughout the coding stage, to ensure consistency in categorizing the issue of complaints, 3 researchers (JL, SCH, and JDM) discussed to eliminate divergence.

## Results

### Search Results

During the process of synthesizing identified literature, 35 studies were excluded for various reasons, including inability to distinguish between positive and negative comments [2,39], fuzzy quantity [11,16,41], channels of complaints [40,42], or only the number of high-frequency words of complaints mentioned [15,43]. Ultimately, 17 publications were identified as eligible. A wide range of data sources were represented, including (1) PRWs (10/17, 59%), such as RateMDs and China’s Good Doctor website; (2) government-managed health websites, such as NHS choices (2/17, 12%); (3) social networking sites (4/17, 24%); and (4) national online surveys (1/17, 6%). Of the 4 articles referring to social networking sites, 2 included data captured from tweets, whereas one was from Google+ reviews and one from Facebook.

### Descriptive and Methodological Data

Through our analysis, we found that pertinent articles have emerged since 2012, with a steady increase observed ever since. Most of the research reported in the analyzed studies focused on PRWs or tweets examining country contexts, such as the United States, the United Kingdom, Germany, China, and Canada. From the data collected, it was identified that 10 articles focused on the United States (59%); 4 on the United Kingdom (24%); and 1 article each on China, Germany, and Canada. Most studies did not screen the departments in which the complainant arose (13/17, 76%), whereas 4 articles (24%) paid attention to complaints received from those dealing with a specific illness or encountering specific medical services. The number of complaints reported (or listed in the thematic analysis) in each study varied widely (average 6543, SD 15,547, range 36-57,028, and median 480).

It was noted that the 17 articles included in our sample had different classification criteria, theme terms, and granularity of complaints. Among them, 6 articles (35%) classified complaints based on published categorization schemes, whereas 9 articles (53%) generated their own coding framework from scratch. The coding framework of 2 articles (12%) came from data source organizations. For example, Zhang et al [9] classified complaints according to the stages of medical consultation, which resulted in a 3-layer classification of stages, including medical consultation, diagnosis and treatment processes, and specific complaint attributions. Emmert et al [44] classified complaints according to the object being complained about and generated a 2-layer taxonomy, referring to both the object and specific complaint attributions. We assessed the quality of the included studies against interrater reliability/performance measures; number of layers; number of codes used, and if there were any definitions or descriptions; and examples provided, as shown in Table 1. The results indicated that the quality of studies varied: 8 did not report any classification measurement results and 4 studies used less than 6 codes, overall, whereas 15 did not provide definitions. A total of 13 studies did not provide examples of each theme/class. Thus, the taxonomy for patients’ online complaints is unstandardized, and it is deemed difficult to identify consistent problems arising in patient care.

**Table 1:** A breakdown of descriptive and methodological data.

Article	Country	Health care settings	Data sources	Complaints reported, n	Source of coding frame	Complaints coded by	Classification quality				
							IRR^a^/performance measure	Layers, n	Codes used, n	Definitions or descriptions present	Examples present
Alemi et al [45] (2012)	United States	Multiple	PRW^b^	307	Survey items	Machine learning algorithms	precision, recall, *F* measure, and area under ROC curve	2	32	No	No
López et al [46] (2012)	United States	Primary care	PRW	263	Developed	Authors	κ^c^	2	24	No	Yes
Lagu et al [8] (2013)	United Kingdom	Multiple	Health website	200	Literature and NHS^d^ Choices prompts	Authors	κ	2	22	No	No
Detz et al [47] (2013)	United States	Primary care	PRW	36	Literature	Authors	NR^e^	2	18	No	No
Emmert et al [44] (2014)	Germany	Multiple	PRW	480	Literature	Authors	κ	2	49	No	No
Greaves et al [48] (2014)	United Kingdom	Multiple	SNS^f^ (tweets)	60	Literature	Authors	κ	3	17	No	No
Macdonald et al [49] (2015)	Canada	Multiple	Dental services	15	Developed	Authors	NR	2	16	No	No
Hawkins et al [39] (2015)	United States	Hospitals	SNS (tweets)	814	Developed	Amazon Mechanical Turk workers/curators	κ	1	10	No	No
Lagu et al [50] (2015)	United States	Multiple	SNS (Facebook)	37	Developed	Two investigators	r_s_^g^	1	4	No	Yes
Trehan et al [51] (2016)	United States	Multiple	PRW	533	Literature	Authors	NR	2	5	No	No
Cunningham and Wells [52] (2017)	United Kingdom	Multiple	Official online survey	1969	Developed	Authors	NR	2	22	No	Yes
James et al [53] (2017)	United States	Multiple	PRW	10,992	Developed	Machine learning algorithms	NR	1	3	No	No
Xu et al [54] (2017)	United States	Multiple	PRW	125	Developed	Authors	NR	1	9	Yes	No
King et al [55] (2017)	United States	Multiple	SNS (Google+ reviews)	34,748	Developed	Customized software	NR	1	2	Yes	No
Brookes and Baker [23] (2017)	United Kingdom	Multiple	Health website (NHS choices)	57,028	Literature	Computer-assisted methods (CQPweb)	NR	2	23	No	Yes
Zhang et al [9] (2018)	China	Multiple	PRW	3012	Developed	Authors	α^h^	3	50	No	No
Emmert et al [56] (2018)	United States	Multiple	PRW	618	Literature	Authors	κ	1	20	No	No

^a^IRR: Interrater reliability.

^b^PRW: physician rating website.

^c^κ: Cohen kappa coefficient.

^d^NHS: National Health Service.

^e^NR: not retrievable.

^f^SNS: social networking service.

^g^r_s_: Spearman correlation.

^h^α: Cronbach alpha.

All papers specified the coders of the complaints; among them, 71% (n=12) were coded by the authors, 12% (n=2) were coded by data curators or investigators, and 18% (n=3) were coded automatically via machine learning techniques. In addition, 9 articles reported intercoder reliability (6 with the Cohen kappa coefficient, 2 with the Spearman correlation or Cronbach alpha, and 1 unspecified).

### Coding Results

In total, across the 17 papers, 326 issue codes were used to code 154,762 complaints. Among them, 36 issue codes incorporating 9602 complaints were not classified into our classification framework for their ambiguous meaning of the category (such as *others*). When classifying all complaint codes, identified from the literature, into our complaint classification code system, we determined that a single code may include a number of new complaint codes that have been assigned individual type codes. The coding results containing concept explanations and issue numbers are provided in Table 2. Patients’ online complaints were seen to fit into the 4 domains, proposed in the a priori framework: (1) prerequisites, (2) patient-centered processes, (3) care environment, and (4) expected outcomes. From the complaints analyzed, it was concluded that patients have the most dissatisfaction with the *patient-centered processes* (101,586/204,363, 49.71%), followed by *prerequisites* (50,563/204,363, 24.74%), and *care environment* (48,563/204,363, 23.76%), with the least satisfied being *expected outcomes* (3651/204,363, 1.79%).

The *prerequisites* domain referred to dissatisfaction with the professional skills and knowledge of the health care provider (48251/204,363, 23.61%) and cooperation between professionals in the medical team (2312/204,363, 1.13%). Among the 4 subcategories, the most common referred to comments about *attributes of the patient-centered professional* (38,314/204,363, 18.75%), which represents the explicit patient-centered personality traits of professionals.

The domain with the most online complaints, *patient-centered processes*, was represented by a number of categories, as shown in Table 2. Within this domain, the greatest number of complaints related to a lack of *open communication of knowledge, personal expertise, and clinical expertise between the patient and the professional* (47,385/204,363, 23.19%). The second category *care plan based on patient's individual needs* collected 40,722 issues (40,722/204,363, 19.93%). The remaining 3 categories contained a small number of complaints, for example, *patient participation as a respected and autonomous individual* was represented by 4.00% (8186/204,363) of total issues. Among the 14 subcategories in this domain, *information, communication, and education* accounted for the majority of complaints, representing 22.80% (46,596/204,363) of total issues reported, whereas no complaints were reported on *patient autonomy*. The most frequently mentioned subtheme in *the care environment* domain was *availability* (28,784/204,363, 14.08%), which represented the timeliness of service and the accessibility of medical staff, facilities, and materials. Common problems mentioned in several articles were lengthy telephone calls made by the physician during consultation and difficulties in patients booking an appointment or seeing a clinician. It is worth noting that issues of *therapeutic environment* emerged in 12 articles. The *expected outcomes* domain contained complaints relating to physical comfort and physical care (3009/204,363, 1.47%), and emotional support for alleviation of anxiety issues (642/204,363, 0.31%).

**Table 2:** Main results of the coding.

Domains, categories, and subcategories			Definition
**Prerequisites (50,563 /204,363, 24.74%)**			
	**Health professionals have appropriate skills and knowledge (48,251/204, 363, 23.61%)**		
		Professional competence (9937/204,363, 4.86%)	Professional competence focuses on the knowledge and skills of the professionals to make decisions and prioritize care and includes competence in physical or technical aspects of care.
		Attributes of the patient-centered professional (38,314/204,363, 18.75%)	The following care attributes are important in professionals' approach to patients: respect, good manners, being polite, good etiquette, sensitive, welcoming, and empathetic.
	**A cohesive and co-operative team of professionals (2312/204,363, 1.13%)**		
		Cooperation among clinicians a priority (2312/204,363, 1.13%)	Patient-centered clinicians are described as being committed and cooperative in an effective team that draws on individuals from different disciplines to complement one another in patient care.
		Differences in perception of role between doctors, nurses, and patients (0/204,363, 0.00%)	Members of the team know exactly the differences in the roles of doctors, nurses, and patients.
**Patient-centered processes (101,586/204,363, 49.71%)**		
	**Participation of the patient as a respected and autonomous individual (8186/204,363, 4.01%)**		
		Respect for patients’ values, preferences, and expressed needs (7446/204,363, 3.64%)	Patient-centered care (PCC) responds precisely to each patient's wants, needs, and preferences.
		Patient as a source of control (370/204,363, 0.18%)	Patients should be given the necessary information and the opportunity to exercise the degree of control they choose over health care decisions that affect them. The health system should be able to accommodate differences in patient preferences and encourage shared decision making.
		Patient’s active involvement and participation (370/204,363, 0.18%)	It gives patients abundant opportunities to be informed and involved in medical decision making and guides and supports those providing care in attending to their patients’ physical and emotional needs and maintaining or improving their quality of life as far as possible.
		Patient autonomy (0/204,363, 0.00%)	Patients direct their lives according to their personal convictions and individual reasons and goals, ultimately to achieve self-governance and self-care.
	**Involvement of family and friends (591/204,363, 0.29%)**		
		Family and friends supported as caregivers (591/204,363, 0.29%)	This dimension of patient-centeredness focuses on accommodating family and friends on whom patients may rely, involving them as appropriate in decision making, supporting them as caregivers, making them welcomed and comfortable in the care delivery setting, and recognizing their needs and contributions.
	**Care plan based on patient’s individual needs (40,722/204,363, 19.93%)**			
		Care customized according to patient needs and values (3101/204,363, 1.52%)	PCC is highly customized, incorporates cultural competence and empowers patient decision making
		Needs are anticipated (462/204,363, 0.23%)	Care plan meets the future needs of patients.
		Coordination and integration of care (35,923/204,363, 17.58%)	The extent to which patient care services are coordinated across people, functions, activities, and sites in a timely manner to maximize the value of services delivered to patients. Patients identified 3 areas in which care coordination can reduce feelings of vulnerability: coordination of clinical care, coordination of ancillary and support services, and coordination of frontline patient care.
		Transition and continuity of care (1236/204,363, 0.60%)	Support patients with their ability to care for themselves after discharge. Meeting patient needs in this respect requires the following: understandable, detailed information regarding medications, physical limitations, dietary needs, etc; coordinate and plan ongoing treatment and services after discharge; and provide information regarding access to clinical, social, physical, and financial support on a continuing basis.
	**Genuine clinician-patient relationship (4702/204,363, 2.30%)**		
		Care based on a continuous healing relationship (4385/204,363, 2.15%)	Patients should receive care whenever they need it and in many forms, not just face-to-face visits. This rule implies that the health care system should be responsive round the clock (24×7) and that access to care should be provided over the internet, by telephone, and by other means in addition to face-to-face visits.
		Clinician-patient relationship (317/204,363, 0.16%)	The effective clinician-patient relationships coming from a healing model, with education and disease management information delivered within the context of the healing relationship.
	**Open communication of knowledge, personal expertise, and clinical expertise between the patient and the professional (47,385/204,363, 23.19%)**		
		Knowledge shared and information flows freely (752/204,363, 0.37%)	Patients should have unfettered access to their own medical information and to clinical knowledge. Clinicians and patients should communicate effectively and share information.
		Information, communication, and education (46,596/204,363, 22.80%)	With respect to their health, people tend to wonder (1) what is wrong (diagnosis) or how to stay well, (2) what is likely to happen and how it will affect them (prognosis), and (3) what can be done to change or manage their prognosis. Common to all such interactions is the desire for trustworthy information (often from an individual clinician that is attentive, responsive, and tailored to an individual’s needs).
		Feedback mechanisms to measure patient experience (37/204,363, 0.02%)	Clinicians can move beyond their individual patients and use survey instruments and other tools that invite patients to report collectively about their clinical experiences.
**The care environment (48,563/204,363, 23.76%)**			
	**System issues (48,563/204,363, 23.76%)**		
		Geographic accessibility (536/204,363, 0.26%)	The physical distance, travel time, and cost from service delivery point to the patient.
		Availability (28,784/204,363, 14.08%)	Having the right type of care available to those who need it, such as hours of operation and waiting times that meet the demands of those who would use care, as well as having the appropriate type of service providers, materials and facilities such as parking, food, and hand hygiene.
		Financial accessibility (2964/204,363, 1.45%)	The relationship between the price of services (in part affected by their costs) and the willingness and ability of users to pay for those services as well as be protected from the economic consequences of health costs.
		Supportive organizational system (8624/204,363, 4.22%)	A system that promotes a philosophy conducive to PCC. Specifically, the system’s managers and employees (usually not clinical experts) create and maintain a responsive, secure, and orderly system on their own or via information systems.
		Therapeutic environment (7655/204,363, 3.75%)	It is the context in which care is delivered. A place quiet, peaceful, neat, clean, and private, if necessary.
**Expected outcomes (3651/204,363, 1.79%)**			
	**Addressing a patient’s physical and emotional needs (3651/204.363, 1.79%)**		
		Physical comfort (3009/204,363, 1.47%)	Attention to physical comfort implies timely, tailored, and expert management of pain, shortness of breath, or other discomforts, with the best possible curative effect. Try the best to avoid unexpected patient events and actively deal with them once they occur.
		Emotional support—alleviation of anxiety (642/204,363, 0.31%)	PCC attends to the anxiety that accompanies every injury and illness, whether because of uncertainty, fear of pain, disability or disfigurement, loneliness, financial impact, or the disease burden on one’s family.

### Country-Specific Differences in Complaints

**Figure 3:**
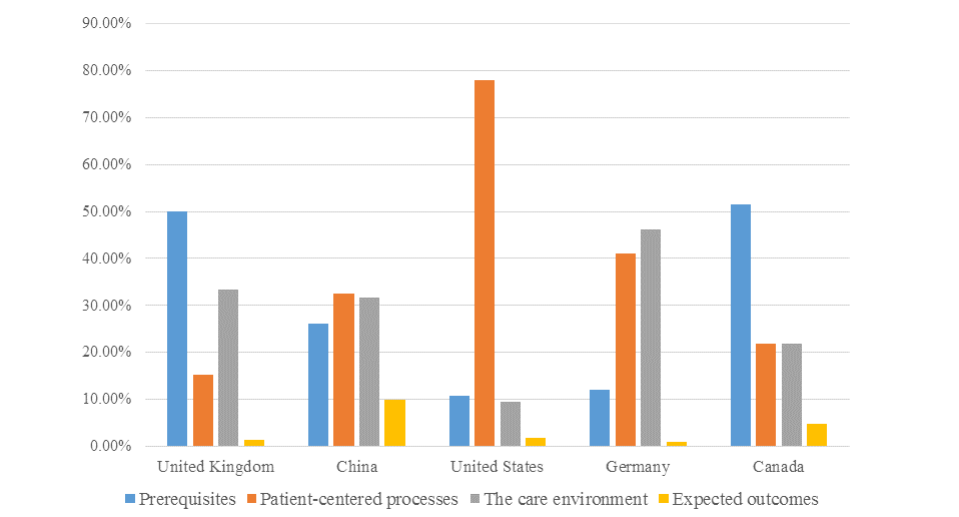
Distribution of 4 domains of online health care complaints across different countries.

We analyzed online complaints from patients based in the United Kingdom, China, the United States, Germany, and Canada because of these being the focus of the studies identified in the literature. The distribution of the 4 domains of online health care complaints across different countries is displayed in Figure 3.

With regard to care aspects that were complained about most frequently, Canadian (33/64, 52%) and British patients expressed the greatest dissatisfaction with *prerequisites* (34,828/69,746/, 49.94%), as compared with those of other nationalities. Referring to Canadian patient experiences of dental practice, dentists’ professional competence caused the greatest dissatisfaction (20/64, 31%), whereas the grievances of British patients to prerequisites were largely related to patients’ discontent over the attributes of the patient-centered professionals, which contributed to 42.74% (29,810 /69,746) of all complaint issues reviewed from British patients. Patients based in the United States attached significant importance to *patient-centered processes* (76,349/97,937, 77.96%), especially relating to information, communication, and education (41,133/97,937, 42.00%). German patients expressed negative comments to *systemic problems* (14,337/31,095), accounting for 46.11% of all complaints identified from German patients. In particular, poor therapeutic environments led to the most-complained-about topic. Chinese patients’ complaints relating to *expected outcomes* represented 9.82% (542/5521) of the total complaints made, far exceeding the sample average (3651/204,363, 1.79%).

In addition to the aforementioned observations, it was identified that UK patients showed special features. First, they expressed dissatisfaction with the dimensions of care that patients in other countries did not complain about. Such dimensions included *family and friends supported as caregivers*, *needs are anticipated*, and *transition and continuity of care*. Second, UK patients expressed far-less-than-average complaints about certain dimensions of care than those in other countries, such as *financial accessibility* (0% vs 1.45%) and *information, communication, and education* (1.94% vs 22.80%). The proportion of complaints from Chinese patients to experts’ professional competence and physical comfort was significantly higher than that of patients from other countries.

## Discussion

### Principal Findings

The results of this systematic review show that there is not yet a widely adopted taxonomy framework for classifying patient complaints made online in various settings. This means that studies have defined and classified complaints differently, resulting in limited comparability between available studies and research contexts [24,25,57]. To eliminate this gap, we have developed an a priori framework of PCC, which can incorporate patient complaints online. The NHS Institute for Innovation and Improvement found that PCC frameworks (eg, Picker framework) are broadly appropriate for “what matters most” to patients [58]. We have further validated the scope of the PCC frameworks using data from patient complaints online. Our research is considered beneficial for identifying gaps in the evidence base for patient experience expressed online, which has been identified as *domains of PCC*.

From the analysis of investigated studies, we have identified that the generic themes of *prerequisites*, *the care environment*, and *the processes* were complained about the most or, put it another way, greatly valued by the majority of patients, regardless of where they came from, and should be deemed priorities for PCC. To be more specific, a practicing clinician should be trained more on these dimensions: attributes of patient-centered professional, information, communication, and education.

Although involvement of family and friends is increasingly viewed as an important component of PCC [1,59-61], patients seldom expressed dissatisfaction with it in our research; apparently far less complained about the low participation of themselves. Compared with technical competence, which constituted a fundamental aspect of health care provision [23], interpersonal attributes of professionals were much more likely to be evaluated by patients; the potential implications of this are twofold: first, consistent with the study by Jia Li et al [26], patients’ needs have different hierarchy, which were firstly stated by Maslow [62], and second, patients have uninformed expectation—“patients are not capable or are reluctant to communicate their expectations” [61], which was validated by Rothenfluh and Schulz [63]. Although there were common concerns across different countries’ patients, variation existed in this study as well. Overall, 5 countries included in our study have different health care systems in terms of health care insurance, drug pricing, physician compensation, etc, and lead to disparities in quality of care, care coordination, and physician education: for example, health care in countries such as the United Kingdom and Canada is publicly financed and the coverage is universal; however, the health coverage of America remains fragmented, with numerous private and public sources, as well as wide gaps in insured rates across the US population [64].

In general, UK patients contributed most of the complaints about the attributes of patient-centered professionals. This finding correlates with that of the NHS Institute for Innovation and Improvement [58]. Besides, British patients had a higher pursuit as they voiced high-level needs such as *involvement of family and friends* (591 issues), *anticipated needs* (462 issues), *feedback mechanisms to measure patient experience* (37 issues), and *emotional support* (642 issues). British patients were also the only nationality to convey discontent over these aspects of care. Given that the NHS has been collecting data on patients’ experience of care for over 10 years [58] and professionals are occasionally accused of being incompetent in satisfying patients’ needs to be treated as a person, eliminating the gap between knowing and doing is crucial. From another perspective, UK patients are assumed to be more *informed* about PCC by virtue of a variety of regular national health and social care surveys carried out in the United Kingdom, with frameworks of several surveys adopting Picker’s PCC principles being available [58,65]; however, this hypothesis awaits further confirmation.

Despite nearly none of the British patients in this study expressing discontent over financial accessibility (0/69,746, 0.00%) and physical outcomes (376/69,746, 0.54%), approximately 10% (542/5521, 9.82%; 441/5521, 7.99%) of Chinese patients’ complaints referred to these issues. Although the coverage by publicly financed health insurance in China is near-universal, out-of-pocket spending per capita represented approximately 32% of total health expenditures in 2014 [64]. Aside from this, high registration fees, the formidable markup by ticket touts operating in health care locations [9], fees related to excessive tests and treatments [26,66,67], and insurance reimbursement obstacles (cross-regional medical treatment) [67] constitute Chinese patients’ financial barriers. It is likely that physician-dominated decision-making [68], inadequate communication, and patient distrust of doctors [66,68,69] have led to dissatisfaction with their physical outcomes.

Looking into the aspects of care that deeply concerned German patients, we find it necessary to improve the therapeutic environment of health care provision, in terms of privacy and entertainment, as well as maintaining a continuous healing relationship through telecommunication and house visits [44]. Given that studies on patient experience in Germany have not taken these vulnerable care aspects into account [70-72], these dissatisfaction factors should be tested in future research.

In this study, patients based in the United States conveyed great dissatisfaction for information, communication, and education (41,133/97,937, 42.00%), as well as coordination and integration of care (33,520/97,937, 34.23%). Previous work on patient-centered communication demonstrated a positive correlation between skilled physician communication and patient satisfaction [73-75]. Utilizing patient-centered communication guidelines and codes of conduct, such as physicians’ humility and communication training for physicians and medical students, may bring better patient experience and diminish patient complaints [70,73-76]. As for coordination and integration of care, as necessitated when patients encounter long waiting times in hospitals or disorganized operations, previous studies have focused on hospital-level care coordination strategies associated with better patient experience [77]. Besides, it is envisaged that information technologies can reduce the need to craft laborious, case-by-case strategies for coordinating patient care [30,78,79].

Our findings suggest that it is feasible to identify gaps in evidence bases for patient experiences, which have been identified as *domains of PCC*. It was observed that differences and commonalities coexist across countries, after applying the proposed taxonomy framework, and we found that there is much leeway for the countries of interest to seek improvement in patient-centeredness.

### Conclusions

Patient complaints online can indicate weaknesses in the health care system through the eyes of the patients’ themselves. The proposed PCC framework can be applied to analyze the complaints under a wide PCC range of conditions, treatments, and countries. This review has shown significant heterogeneity of patients’ online complaints in different countries, attributable to the diversity in culture, health care institution, and health literacy. Further work is required to apply the framework, using a plethora of data sources, to compare with other services, organizations, and countries or within the health care service over time, that is, a longitudinal study.

All RQs, proposed in the Introduction of this paper, were answered through conduction of the systematic review. Despite certain studies classifying patient complaints online, none were found to include or adopt a credible taxonomy framework. The proposed PCC framework aligns with what patients currently complain about online. By applying the taxonomy, results show that health professionals’ skills and knowledge, open communication of knowledge, and system issues of PCC constitute the focus of online complaints made by patients. In addition, the differences in patient complaints in a multicountry context are discussed.

### Limitations

As always, there are several limitations to this study. First, it was based on searches in merely 3 databases and focused only on currently available peer-reviewed literature; for this reason, we may have missed information in the gray literature. Second, regarding the small number of included articles, because of artificial screening, and the resulting relatively small sample size, our conclusions, especially the country-specific ones, may not be free of overgeneralizations and missing targets, even by taking into account the “community of common destiny.” Finally, our interpretation of the concepts and scope of the various categories of complaints included in the article may not be fully consistent with the authors of the included papers, especially if the explanations or quotes were not given. Considering the limits to the time, space and researcher resources of this study, it is, nonetheless, a worthy trial that merits further exploration.

## Appendix

Multimedia Appendix 1 Search strategy.

## Abbreviations

NHS: National Health Service
PCC: patient-centered care
PRW: physician rating website
RQ: research question
