# Supplementary material for: What Do Patients Complain About Online: A Systematic Review and Taxonomy Framework Based on Patient Centeredness
Source: J Med Internet Res. 2019 Aug 7;21(8):e14634. doi: 10.2196/14634 (PMC6702801; doi:10.2196/14634)
Supplement: Multimedia Appendix 1 [file jmir_v21i8e14634_app1.pdf]

Search strategy

Pubmed 1/7/2018

1	patient*[Title] OR consumer*[Title]	1664910
2	(comment*[Title] OR rating*[Title] OR suggestion*[Title] OR complaint*[Title] OR review*[Title] OR feedback*[Title]) AND (experience[Title/abstract] OR quality[Title/abstract] OR safety[Title/abstract] OR satisfaction[Title/Abstract] OR patient center*[Title/Abstract])	89057
3	"Health Care Quality, Access, and Evaluation"[Mesh]	6745771
4	(online[Title/Abstract] OR free text[Title/Abstract] OR narrative*[Title/Abstract] OR social media[Title/Abstract] OR ehealth[Title/Abstract] OR virtual[Title/Abstract] OR internet[Title/Abstract] OR facebook[Title/Abstract] OR twitter*[Title/Abstract]	203947
5	(#2 OR #3) AND #4 AND #1	9274
6	("2000"[Date - Publication]:"2018/06/30"[Date - Publication] and #5	8937

Web of science 1/7/2018

1	TI=(“patient*” OR “consumer*”)	2965051
2	TI=(“comment*” OR “rating*”OR “suggestion*” OR “complaint*” OR “review*”OR “feedback*”) AND TS=(“experience” OR “quality” OR “safety” OR “satisfaction” OR “patient center*”)	178244
3	TS=(“online” OR “free text” OR “narratives” OR “social media” OR “ehealth” OR “virtual” OR “internet” OR “twitter*” OR “facebook”)	1711172
4	#2 AND #3 AND #1<br>TIME SPAN=2000-2018	943

Scopus 1/7/2018 (1156 documents)

(TITLE("patient*") OR TITLE ("consumer*")) AND (TITLE ("comment*") OR TITLE ("rating*") OR TITLE ("suggestion*") OR TITLE ("complaint*") OR TITLE ("review*") OR TITLE ("feedback*")) AND (TITLE-ABS-KEY ("experience") OR TITLE-ABS-KEY ("quality") OR TITLE-ABS-KEY ("safety") OR TITLE-ABS-KEY ("satisfaction") OR TITLE-ABS-KEY ("patient center*")) AND (TITLE-ABS-KEY ("online") OR TITLE-ABS-KEY ("free text") OR TITLE-ABS-KEY ("narratives") OR TITLE-ABS-KEY ("social media") OR TITLE-ABS-KEY ("ehealth") OR TITLE-ABS-KEY ("virtual") OR TITLE-ABS-KEY ("internet") OR TITLE-ABS-KEY ("facebook") OR TITLE-ABS-KEY ("twitter*")) AND PUBYEAR > 1999
